# The effect of credibility assessment techniques on consistency and subsequent memory for the truth

**DOI:** 10.3389/fpsyg.2023.1184055

**Published:** 2023-06-26

**Authors:** Rachel E. Dianiska, Christian A. Meissner

**Affiliations:** ^1^Department of Psychological Science, University of California, Irvine, Irvine, CA, United States; ^2^Department of Psychology, Iowa State University, Ames, IA, United States

**Keywords:** lying, memory accuracy, consistency, credibility assessment, evidence-based interviewing

## Abstract

Repeated interviews are common during an investigation, and perceived consistency between multiple statements is associated with an interviewee’s credibility. Furthermore, research has shown that the act of lying can affect a person’s memory for what truthfully occurred. The current study assessed the influence of lying on memory during initial and repeated interviews, as well as how an interviewer’s approach might affect between-statement consistency for true and false statements. Participants performed a scavenger hunt at two sets of buildings on a university campus and then were either dismissed or interviewed (with a Reverse Order instruction or a Structured Interview) about their activities. Participants chose one set to tell the truth about and then created a lie about activities in another area of campus that had not been visited. One week later, all participants provided a second free recall statement about their activities during the scavenger hunt, and then a final truthful description of both areas that were visited during the scavenger hunt. Truthfully rehearsed experiences were associated with more accurate recall of information learned during the scavenger hunt as well as more consistent and more detailed statements. The Structured Interview led to initially more detailed statements, but more inconsistencies in the form of omissions.

## 1. Introduction

Investigators routinely conduct repeated interviews with the same suspect during an investigation (Kassin et al., 2007). In this context, alterations to a statement, regardless of the intention behind them, are often used to question the reliability of a witness’s statement (Brewer et al., 1999) and to identify a subject who may be providing a false statement (Granhag and Strömwall, 2001). Repeated interviews therein pose a quandary for both innocent and guilty suspects.

Innocent suspects may choose to be strategically forthcoming and cooperative with an investigation when telling the truth (Hartwig et al., 2007; Granhag and Hartwig, 2008). When interviewed again at a later time, inconsistencies might appear if the individual were to offer new information that was not provided in a previous statement (i.e., a *reminiscent* detail), or if they failed to recall information that was provided previously (i.e., a *forgotten* detail). Though reminiscence and forgetting reflect natural underlying cognitive processes that can arise as a result of repeated retrieval (Ballard, 1913), such inconsistencies can lead an investigator to question a truth-teller’s credibility.

Guilty suspects, on the other hand, will need to remember a previously provided false statement in order to maintain consistency across interviews. False statements that a guilty suspect can provide include false descriptions (or fabrications), and false denials of events (e.g., simple denials or simulated amnesia; Otgaar and Baker, 2018). In the former, a suspect may describe an event or an experience differently than how it actually occurred or describe an event that never occurred. Lying, in this instance, requires the suspect to confabulate details to create a plausible account. Alternatively, the suspect may lie by falsely denying that an event occurred, despite the fact that the event did take place. Psychological research has shown that the type of false statement that is provided can carry implications for one’s ability to remember that lie, and that the act of lying can change a person’s memory for the truth (Otgaar and Baker, 2018; Dianiska et al., 2019; Battista et al., 2020; Dianiska and Meissner, 2022). For example, denying or simulating amnesia can lead to more errors of omission, while lying by describing can lead to more errors of commission. As such, the manner in which a guilty suspect provides a false statement could influence not only their ability to appear credible (i.e., consistent) on subsequent interviews, but also their memory for what truthfully occurred.

False descriptions and false denials differ in the extent to which effortful, constructive mental processing is required. As a result, these two types of lies tend to differentially affect both accurate memory and false memory (Vieira and Lane, 2013; Battista et al., 2020). Lies that are told via false description are more likely to be correctly remembered due to the constructive processes involved in generating the descriptions (Riesthuis et al., 2020; Battista et al., 2021; Dianiska and Meissner, 2022). Providing a brief false denial, on the other hand, requires less effort to produce and is therefore more easily forgotten (Otgaar and Baker, 2018; Dianiska et al., 2019). In addition to denials being less effortful, poor memory for denials may also be due to an inhibitory mechanism (Anderson and Neely, 1996; Debey et al., 2015). However, memory for false denials can improve when the denials are repeated (Vieira and Lane, 2013; Dianiska and Meissner, 2022). Due to the constructive processes involved in lying by describing, false descriptions can paradoxically be more likely to be *misremembered* as the truth should the act of generating a description as a lie (rather than as a truth) be forgotten (Polage, 2012; Vieira and Lane, 2013). This process is likely a result of source misattribution, where one mistakes the origin of that description (Johnson et al., 1993). In this case, the content of the lie is retained, but the reason for its generation (e.g., to tell a lie) is not.

### 1.1. Memory, consistency, and perceived credibility

Relying on consistency as an indicator of truthfulness can negatively affect innocent suspects who seek to be cooperative with an interviewer. Truth-tellers’ statements will be grounded in their memory for an event, and the reconstructive nature of memory increases the likelihood of errors (Bartlett, 1932). Should an innocent person provide an initially mistaken alibi statement due to faulty memory and come to realize their error, any attempt to correct their statement by providing contradictory information might lead to suspicion as a result of that inconsistency (Crozier et al., 2017). As such, unwarranted mistrust of an inconsistent (but innocent) alibi provider could potentially redirect the course of an investigation away from pursuing a different suspect. Investigators must consider not only the presence and type of an inconsistency in a statement, but also the role of memory recall inherent to producing that statement. Although some statement-enhancing questioning techniques strategically support and capitalize on an interviewee’s memory, the impact of such tactics have not yet been fully assessed with respect to possible misattributions of deception and guilt due to inconsistencies across statements.

Regardless of guilt, the interaction between lying and memory has implications for a suspect’s experience with the criminal justice system. For instance, whether guilty suspects are able to maintain their false narratives over time could have significant downstream consequences that lead to their conviction. On a subsequent interview, the ability to remember (and repeat; Granhag and Strömwall, 1999) what was said in an initial interview is extremely important given the common perception that inconsistency is associated with deception (Vredeveldt et al., 2014).

### 1.2. Consistency across repeated interviews for truthful and deceptive accounts

Though inconsistencies are often treated by laypeople and professionals as indicators of deception, research suggests that it is the *type* of inconsistency that is a more important indicator of deception, rather than inconsistency itself (Fisher et al., 2013; Vredeveldt et al., 2014). Across repeated interviews, engaging in varied retrieval can contribute to the reminiscence of details not previously reported. Gilbert and Fisher (2006) examined the effects of varied retrieval across a repeated interview context on inconsistencies in the form of contradictions, reminiscences, and omissions. Varying the retrieval cues between two event recall opportunities increased the amount of reminiscent information reported and decreased the number of items that were omitted on the second event recall. The amount of consistent and contradictory items that were recalled were similar. Gilbert and Fisher also found that the accuracy of inconsistent-reminiscent and inconsistent-omitted details was fairly high (87 and 93%, respectively). Consistent details, however, were still associated with the highest accuracy (95%). Few contradictory details were reported overall, but when they were reported, they were associated with low accuracy (49%).

For guilty suspects, there are different types of (in)consistency that can induce suspicion, including the perceived consistency within a suspect’s statement and across multiple statements. Inconsistencies can also arise between statements elicited from multiple suspects, or between a suspect’s statement and the available evidence. Interviewers can use strategic questioning approaches to encourage the production of some inconsistencies to facilitate credibility assessment. Consistency across statements has been suggested to be indicative of liars who have rehearsed their statement (Vrij et al., 2009; Masip et al., 2016), liars who underestimate the extent to which forgetting occurs (i.e., stability bias; Harvey et al., 2017a,b), and/or liars who deliberately repeat the same statement given previously to avoid being exposed (Granhag and Strömwall, 1999). However, manipulating the way in which a suspect provides a statement can prevent a liar from using a “repeat” strategy to appear consistent.

Liars are likely to be *inconsistent* when faced with varied retrieval, such as when they must report an event differently between multiple interviews. For example, (Leins et al., 2012) asked liars and truth-tellers to describe their activities in an initial interview either verbally, by providing an initial free recall and then answering specific questions from an interview, or pictorially, by producing a sketch drawing of the task room and the location of as many items as possible. After a 10-min delay, participants provided the interviewer with an additional statement about their activities in the same or different reporting method. Truth-tellers were more consistent than liars (when only items that were *contradictory* were compared to items that were consistent); however, liars were even less consistent when the retrieval method differed between interviews.

### 1.3. Evidence-based interviewing techniques and consistency

Researchers and practitioners have advocated for the use of evidence-based interviewing techniques to increase cooperation and disclosure of information in investigative interviewing (Vrij et al., 2014; Vrij and Granhag, 2014; Meissner et al., 2017; Brandon et al., 2018). Such interviewing tactics have been assessed as both tools to improve the quality of an interviewee’s memory report as well as to magnify differences in verbal content between liars and truth-tellers that aid lie detection (Vrij, 2015), particularly given that the most successful training protocols for lie detection and credibility assessment focus on such verbal content (see Hauch et al., 2016). Examples include eye closure instructions (Perfect et al., 2008), mental context reinstatement (Smith and Vela, 2001), recalling an event in reverse temporal order (Vrij et al., 2008), and asking subjects to sketch along with their statement (Deeb et al., 2022).

The primary goal of these techniques is to increase the amount of information obtained from an interview without a commensurate decrease in accuracy. Techniques that encourage a speaker to elaborate, however, can sometimes lead to the provision of information that may not be true (or information that they might be unsure of; Koriat and Goldsmith, 1996) due to an interviewee reporting incorrect information (i.e., errors in describing a witnessed detail) or confabulating novel details (i.e., errors in describing unwitnessed details). Should an interviewee report such erroneous information on a subsequent interview (or amend a prior statement to correct an error), an interviewer could note a difference between the two statements and infer deception on the part of the subject. However, an error that persists could become incorporated into the subject’s memory for what truthfully occurred (e.g., self-generated misinformation; Pickel, 2004), irreparably affecting their credibility if the information is revealed to be inaccurate.

One tactic that has been evaluated as a credibility assessment tool is a *reverse-order recall* instruction (Vrij et al., 2008; Evans et al., 2013). After an interviewee has provided an initial free narrative, they are asked to recall the event once more in reverse chronological order. Recalling an event from multiple retrieval perspectives, in particular one that is counter to an initial schema-guided retrieval attempt (Geiselman and Callot, 1990), can allow for a previously inaccessible memory trace to be accessed and therein increase the amount of information reported. Asking for an event description in reverse-order increases cognitive load more so for liars than truth-tellers, thereby magnifying discernible verbal and nonverbal behaviors between the two (Vrij et al., 2008; Evans et al., 2013). However, when compared with a request for an open-ended narrative, recalling an event in reverse-order can sometimes increase confabulations and decrease overall statement accuracy (Dando et al., 2011). Errors that are produced as a result of a reverse-order instruction could persist across repeated interviews, leading to further consequences for interviewees with respect to perceived inconsistency (Fisher et al., 2013).

Interviewers’ use of reverse-order recall can induce inconsistencies in both liars and truth-tellers (Gilbert and Fisher, 2006; Hudson et al., 2019). Hudson and colleagues examined consistency between two statements provided in close succession to each other. When a reverse-order recall instruction was administered, both liars and truth-tellers provided more omissions and fewer repetitions. Overall, truth-tellers provided more details across the two interviews, and specifically more reminiscent details during a second interview than did liars. Liars, in contrast, made significantly more omissions when a reverse-order recall instruction was administered during an interview, compared to when a chronological order recall instruction was administered.

### 1.4. Present study

**Figure 1:**
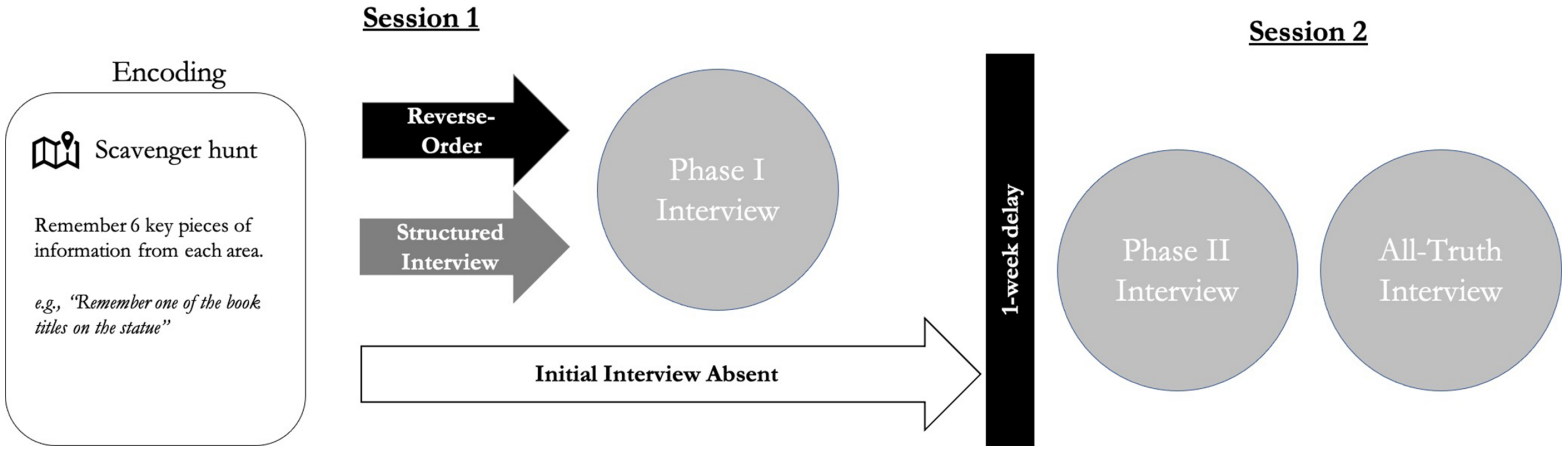
Experiment procedure.

The purpose of the present study was to examine the potential for evidence-based interviewing tactics to foster the generation of inconsistencies across multiple interviews, as well as the potential detrimental influence of providing a false statement on memory for the truth. We used a behavioral paradigm in which participants completed a series of complex tasks prior to being interviewed about them (see Figure 1). Participants experienced two distinct events (scavenger hunt tasks in two areas of a university campus) and then had the opportunity to choose which event they would rehearse truthfully. They then created a false description about activities about a third event, a set of building that had not been visited. Participants were then randomly assigned to be initially interviewed using one of two forensic interview protocols (a Structured Interview or a Reverse-Order recall instruction), or to not complete an initial interview. Seven days later, all participants returned for a second session, during which time they were interviewed about their activities the week prior. In the second interview, participants were provided an open-ended prompt to freely recall each of the two events that they experienced in the first session, describing each event truthfully (or deceptively). Lastly, participants provided a final truthful description of the lied-about event, as well as a final account of the truthfully rehearsed event.

We examined how lying on a prior interview affects one’s memory for what truthfully occurred, and how interviewing techniques might affect the consistency of information reported across repeated interviews. It was hypothesized that fewer correct details would be recalled about a lied-about event during the final all-truth statement, compared to events that were rehearsed truthfully. We further expected that truthfully-described events would be associated with an overall greater amount of detail, and associated with greater consistency and/or inconsistencies in the form of reminiscences. With respect to the Reverse Order instruction, we predicted that this interviewing technique would lead to more overall total statement detail, as well as more inconsistencies in the form of omissions (i.e., details present in an initial statement, but not repeated during the second).

## 2. Methods

### 2.1. Pre-registration

This study was pre-registered on OSF (https://doi.org/10.17605/OSF.IO/AJ296) to include sample size, methods, primary hypotheses, and planned analyses.

### 2.2. Participants

A total of 112 participants (56 female) were recruited from a large Midwestern state university, and 105 completed the full experiment (*n* = 7 dropped out between Session 1 and Session 2). Data from six additional participants were excluded for not complying with interview instructions for either Phase I or Phase II interviews. Thus, the final sample included slightly uneven cells for Interview Absent (*n* = 29), Reverse Order (*n* = 32), and Structured Interview (*n* = 38) conditions. Participants age varied between 18 and 28 years of age (*M* = 19.38, *SD* = 1.39).

Due to University closure in the Spring of 2020 in response to the COVID-19 virus, data collection ended prematurely. The target sample of 144 research participants (*n* = 48 per group) specified in the pre-registration would provide sufficient power to detect a relatively small within-between interaction effect size (*f* = 0.15) with power of 0.90 (Faul et al., 2009). This effect size was chosen based on prior work demonstrating differences in consistency for liars and truth-tellers across repeated interviews (e.g., *f* = 0.31 in Leins et al., 2012) and robust increases in total detail following strategic interviewing techniques (e.g., *f* = 0.20 when comparing chronological recall and reverse order recall in Hudson et al., 2019). To appropriately power an interaction between Veracity and Interview Technique, a more conservative effect size was used (*f* = 0.15) than has been observed in prior work. Data analyzed and presented here represent those collected prior to the university closure in March of 2020. Because of ongoing institutional changes with respect to research procedures and the permanent closure of areas of the university campus included in the current study, the remaining participants needed to fulfill the proposed target sample were unable to be collected. Had the power analysis been conducted to be less conservative (0.80), the current sample size would have been sufficient to detect the anticipated effect size.

### 2.3. Design

A 3 (*Initial Interview Technique*: Absent, Reverse-Order, Structured Interview) × 2 (*Veracity*: Lie, Truth) × 2 (*Interview Time*: Phase I, Phase II) mixed design was used. Initial Interview Technique was manipulated between-participants, while Veracity and Interview Time were manipulated within-participants.

### 2.4. Procedure

Participants completed two sessions conducted 1 week apart (see Figure 1). The first session comprised the Encoding Phase and the Phase I Interview (for initially-interviewed conditions). Participants visited four buildings (two pairs of buildings total) on the university campus and completed a scavenger hunt for information within each building. After completing the scavenger hunt, some participants were interviewed about their activities (Reverse Order and Structured Interview conditions) and some were dismissed from the session (Absent condition). Before being interviewed, participants were instructed that they would truthfully tell the interviewer about one pair of buildings of their choice; they would not discuss the other pair of buildings they visited, and instead were instructed to lie about a specific set of buildings that were not visited during the experiment.

#### 2.4.1. Encoding phase

Upon arrival to the session, participants received instructions and provided informed consent to complete the experiment. Before beginning the Encoding Phase, participants completed a brief survey assessing their familiarity with six buildings on the University campus on scale from 1 (I have never been there/Not familiar) to 7 (I know the ins and outs of the building/Extremely familiar). During the Encoding Phase, participants completed what they believed to be a study assessing people’s memory for previously performed activities. Participants received instructions that they would be going to different buildings on the university campus and performing a scavenger hunt at each one. Participants then navigated to two “areas” of campus (i.e., two buildings near each other) and completed a series of brief tasks at each one. Throughout the course of the Encoding Phase, participants were tasked to remember six key pieces of information that they learned in each area. Three versions of the scavenger hunt were created, such that each pair of buildings was equally presented to participants as the first area or second area to which they navigated. All tasks and instructions for the scavenger hunt can be found on the OSF repository.

When participants arrived back to the lab, those in the Interview-Absent condition were dismissed and asked to return 1 week later to complete Phase II. Those in the Interview-Present conditions (Reverse-Order, Structured Interview) received instructions for the initial interview phase. Participants were told that they would be interviewed about their actions after leaving the lab. For the interview, they were asked to tell the truth about one area of campus (meaning one “pair” of buildings) and lie about another area of campus. The participants could choose to tell the truth about either the first pair of buildings or the second pair of buildings that they visited but were instructed that they must lie about another, pre-specified set of buildings.

For the lied-about event, participants were instructed that they would create a detailed, believable cover story. Participants were provided with a worksheet with minimal information about the buildings they were tasked with lying about (taken from the public access building information available on the University’s Facilities Planning and Management website; see OSF) and given 5–6 min to write down details that could be provided in their narratives. To motivate participants to lie well during the task, participants were told that their interviews would be evaluated by other people after the session has concluded, and the person who was judged to be most believable will win a $25 reward. After preparing for the interview, the experimenter confirmed that the participant understood the instructions for the interview task and then left the room to notify the interviewer.

#### 2.4.2. Phase I interview

The participants interviewed in Phase I were randomly assigned to be interviewed with a Reverse Order Instruction or a Structured Interview. Interviews always began by asking for an initial open-ended narrative of participants’ activities at the first area of campus, and then an open-ended narrative for the second area of campus.

In the *Reverse Order* condition, the interviewer followed up the initial request by asking the participant interviewee to recall their activities in the two areas once again in reverse chronological order, beginning from the last temporal detail that they provide for each area. In the *Structured Interview* condition, the interviewer followed up the initial request by asking three probing questions about details the participant had mentioned for each area of campus.

After the conclusion of the interview, participants completed a brief post-interview questionnaire. In addition to demographic information, participants reflected on how well they remembered the tasks that they had completed, what strategy they used to select which event to truthfully describe, how motivated they were to be perceived as truthful, if they did anything in particular to convince the interviewer that they were telling the truth, how comfortable they are with lying in everyday life, as well as global perceptions of the interviewer.

#### 2.4.3. Phase II interview

One week later, all participants (Interview-Absent, Reverse-Order, Structured Interview) returned to the lab to complete the Phase II interview. At the beginning of the session, the experimenter informed all participants that they would be interviewed (for the first time, for Interview-Absent participants; or again, for Reverse-Order and Structured Interview participants) about their activities during the first session of the experiment. Participants were asked to provide a free recall narrative of the two areas of campus that they visited the week prior. At this time, Interview-Absent conditions were given the same lie-truth instructions and cover story preparation time as participants who were interviewed in Phase I. All other participants (Reverse-Order and Structured Interview participants) were instructed to continue to respond truthfully or deceptively for each area of campus as they had in Phase I. During the Phase II interview, the interviewer requested only an open-ended narrative from participants recalling as much information as possible about their activities in both areas of campus.

#### 2.4.4. Final all-truth interview

After describing the two areas of campus truthfully and deceptively, the interviewer informed participants that it was known they were told to lie about their activities in the previous session. Therefore, the participant’s final experimental task was to describe both events as they had *actually* occurred. In addition to providing a third and final statement about their truthfully rehearsed event, participants were told to cease responding deceptively (about their chosen, lied-about event) and to describe their activities in the second, visited area truthfully and in as much detail as possible. This final truthful recall allowed us to assess the influence of having previously lied about an event on subsequent recall of the experienced details.

At the conclusion of the Phase II interview, participants completed a similar post-experiment questionnaire as in the earlier session. These questions reflected overall memory for the tasks in Phase I, strategy use, motivation, comfort with lying in everyday life, and perceptions of the interviewer and the interview experience. Further, they were asked to what extent they expected to be interviewed again, as well as the extent to which they expected the second half of the interview (i.e., the Final All-Truth interview) and how difficult was it to truthfully recall their activities from the first session. Participants who completed an initial interview were also asked the extent to which they attempted to repeat everything they had said previously about their activities during Phase I (i.e., to be consistent) and to what extent they attempted to provide new information about the first and second areas they visited. For the Interview-Absent participants, this questionnaire contained the same questions as the post-Phase I interview questionnaire. Finally, participants completed a cued-recall test for the details that they were tasked to remember during Phase I. Before being debriefed, participants were asked whether they had rehearsed their story or discussed the experiment with anyone since completing Phase I.

#### 2.4.5. Coding of interview statements

Video recorded interviews for each phase (Phase I, Phase II, All-Truth) were coded for subsequent analysis. For Phase I interviews, trained research assistants coded details that were present during the initial chronological narrative that were also *repeated* after the reverse order instruction or structured interview probes were administered, as well as details that were *added* to participant statements after the instruction or probing questions were implemented. A Total Phase I unique details measure was computed by summing: (i) consistent pre- and post-tactic details, and (ii) new details post-tactic. For participants who were not interviewed during Phase I, the same coding scheme was applied for their Phase II interviews.

For all other participants, Phase II interviews were coded for details that were: (i) repeated between Phase I and Phase II (*consistent* details); (ii) contradictory to details provided during Phase I (*inconsistent-contradiction* details); (iii) added during Phase II but not reported during Phase I (*inconsistent-reminiscent* details); and (iv) failed to be provided during Phase II but were provided at Phase I (*inconsistent-omitted* details). Statements from participants who were interviewed in Phase I and Phase II were coded by two coders for the volume of information provided and the consistency of details that were provided. Inter-rater reliability was high (*r*’s > 0.93 for each described area). We computed the total amount of detail provided at Phase I and Phase II, as well as the amount of consistent details, omitted details, and new details reported across statements.

During the final All-Truth interview, participants were instructed to provide a final truthful statement for both areas of campus they had actually visited during Phase I. These all-truthful statements were then coded for the amount of detail provided for both areas of campus–one that they had rehearsed truthfully in the earlier Phase I and Phase II interviews, and one that they had lied about by describing their activities in an alternate area of campus. We assessed accuracy with respect to participants’ freely recalled statements, and with respect to their performance on the cued recall test at the end of Phase II. If participants mentioned a detail they were tasked to remember during their all-truth narrative, the detail was coded as a “1” if it was present and accurate in the statement (e.g., correctly recalling “1926” as the year a fountain was installed). The same was true if participants correctly answered the cued recall question on the final test. A score of “0” for a detail was given for inaccurate details (e.g., an incorrect year), non-specific details (e.g., saying they were told to remember a year, but not providing the year), or when the participant did not mention or said they could not remember the item. Accurate details per area thus ranged from 0 to 6 details, and from these a proportion of accurate details was computed based on the number of details that were mentioned (note: the pattern of results does not change when the proportion of all potential key details are included, rather than just those details mentioned correctly or incorrectly).

## 3. Results

All materials and data are hosted on OSF.^1^ Descriptive statistics from each condition across all measures can be found in Table 1. The following results are distinguished by whether they were pre-registered or exploratory. We first assess the effects of the veracity of a statement and the presence/type of interview tactic used to elicit an initial narrative on participants’ ability to correctly recall information learned during the scavenger hunt. Next, we examine the effects of statement veracity and the type of interview technique used on the amount of information provided during initial and subsequent interviews, and then specifically consistent or inconsistent details provided therein. Finally, we explore differences in the amount of detail provided in participants’ initial recall statements of each event.

**Table 1:** Descriptive statistics for all measures.

	Initial interview absent		Reverse-order		Structured interview	
	Mean	SE	Mean	SE	Mean	SE
*Phase II–Final recall accuracy*						
Prev Lie	0.33	0.06	0.39	0.06	0.26	0.04
Prev Truth	0.40	0.06	0.51	0.06	0.46	0.05
*Phase II–Cued recall accuracy*						
Prev Lie	0.58	0.04	0.65	0.05	0.54	0.04
Prev Truth	0.66	0.05	0.71	0.04	0.69	0.03
*Phase II–Consistency*						
Lie	–	–	20.57	1.11	13.83	1.00
Truth	–	–	23.27	1.18	16.48	1.07
*Phase II–Omissions*						
Lie	–	–	10.30	1.24	15.62	1.12
Truth	–	–	8.68	1.12	16.88	1.02
*Phase II–reminiscence*						
Lie	–	–	3.92	0.65	4.86	0.59
Truth	–	–	5.06	0.54	4.64	0.49
*Phase I–Total detail*						
Lie	–	–	20.88	2.05	37.87	3.47
Truth	–	–	23.22	2.13	40.71	2.91
*Phase II–Total detail*						
Lie	21.00	1.80	18.88	1.96	23.42	1.99
Truth	25.31	2.07	22.16	2.23	26.32	1.93
*Final all-Truth–Total detail*						
Prev Lie	13.96	0.90	14.84	1.02	14.92	0.94
Prev Truth	14.37	1.06	15.38	0.93	14.17	0.93

### 3.1. Pre-registered analyses

#### 3.1.1. Correct recall on final all-truth interview

**Figure 2:**
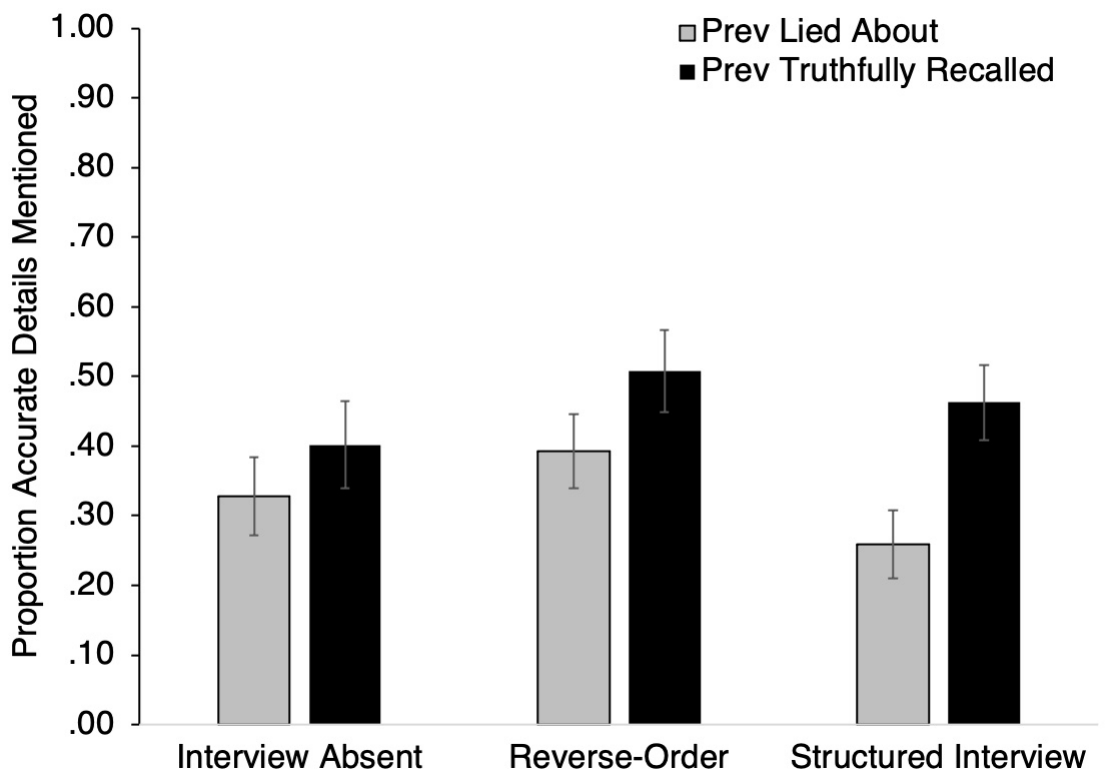
Proportion of key details correctly recalled the final all-truth interview.

At the conclusion of Phase II, participants provided a final, truthful account of their activities involving both areas of campus that they visited during the Encoding Phase. This interview allowed us to assess the influence of having previously recalled an event truthfully vs. deceptively. A 3 (*Initial Interview Technique*: Absent, Reverse-Order, Structured Interview) × 2 (*Veracity*: Lie, Truth) ANOVA was conducted on the proportion of accurate details mentioned in participants’ all-truth interview statements (see Figure 2). As hypothesized, there was a significant main effect of Veracity such that memory for key details was more accurate for areas of campus that participants had previously truthfully recalled (*M* = 0.46, *SE* = 0.03) than areas of campus they had lied about (*M* = 0.33, *SE* = 0.03), *F*(1, 96) = 15.44, *p* < 0.001, *d* = 0.41 [0.21, 0.62]. Neither the main effect of Interview Technique (*F*(2, 96) = 1.13, *p* = 0.33, η_p_^2^ = 0.02) nor the interaction between Initial Interview Technique and Veracity (*F*(2, 96) = 1.37, *p* = 0.26, η_p_^2^ = 0.03) were significant. Performance on the cued recall test was similar and is reported on OSF.

#### 3.1.2. Phase I and phase II total details

A 2 (*Initial Interview Technique*: Structured Interview, Reverse-Order) × 2 (*Veracity*: Lie, Truth) × 2 (*Interview Time*: Phase I, Phase II) ANOVA was conducted on the total number of details present in participant’s statements. There was a significant main effect of Veracity, *F*(1, 68) = 9.28, *p* = 0.003, *d* = 0.37 [0.13, 0.61]; Interview Time, *F*(1, 68) = 67.53, *p* < 0.001, *d* = 0.83 [0.55, 1.10]; and Interview Technique, *F*(1, 68) = 11.62, *p* = 0.001, *d* = 0.82 [0.33, 1.31]. As expected, people provided more details when truthfully describing their activities (*M* = 28.10, *SE* = 1.62) than when creating false descriptions of their activities *(M* = 25.26, *SE* = 1.69). Further, participants provided more detailed statements during Phase I (*M* = 30.67, *SE* = 1.90) compared to Phase II *(M* = 22.69, *SE* = 1.37). However, participants provided more detailed statements when they were interviewed with a Structured Interview script (*M* = 32.08, *SE* = 2.14) compared with a Reverse Order instruction (*M* = 21.28, *SE* = 2.33), as we predicted. Importantly, the main effects of Interview Time and Initial Interview Technique were qualified by a significant interaction, *F*(1, 68) = 44.09, *p* < 0.001, η_p_^2^ = 0.39. While there was a significant decrease in the amount of information recalled from Phase I to Phase II for both conditions, this difference was much greater in the Structured Interview condition (*t*(37) = 8.24, *p* < 0.001, *d* = 1.34 [0.89, 1.77]) than in the Reverse Order condition (*t*(31) = 3.95, *p* < 0.001, *d* = 0.70 [0.31, 1.08]). No other main effects or interactions were significant, *F*’s < 0.25, *p*’s > 0.62.

#### 3.1.3. Between-statement-consistency

**Figure 3:**
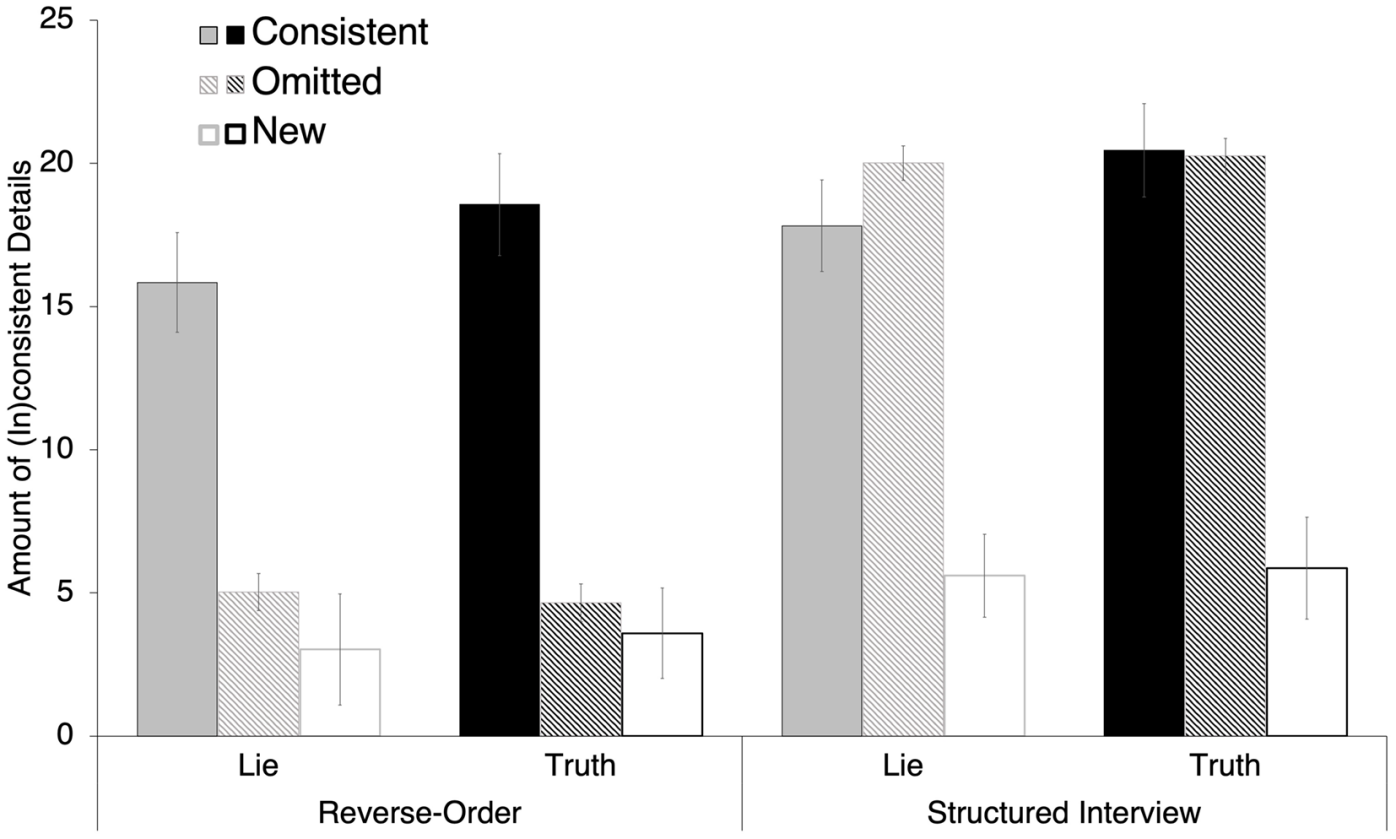
Number of details that were consistent or inconsistent between Phase I and Phase II interviews.

A 2 (*Initial Interview Technique*: Reverse-Order, Structured Interview) × 2 (*Veracity*: Lie, Truth) mixed ANOVA was conducted on the number of details *consistently* provided between Phase I and Phase II (see Figure 3, solid gray and black bars). There was a significant main effect of Veracity, *F*(1, 68) = 11.37, *p* < 0.001, *d* = 0.41 [0.16, 0.65]). People provided more consistent details between Phase I and Phase II when describing their truthfully rehearsed event (*M* = 19.59, *SE* = 1.20) than when describing their lied-about event (*M* = 16.91, *SE* = 1.18). However, there was no main effect of Interview Technique (*F*(1, 68) = 0.73, *p* = 0.40, η_p_^2^ = 0.01, nor an interaction between Veracity and Interview Technique (*F*(1, 68) = 0.06, *p* = 0.96, η_p_^2^ < 0.01).

With respect to inconsistency, we examined differences in omissions and new details separately via 2 (*Initial Interview Technique*: Reverse-Order, Structured Interview) × 2 (*Veracity*: Lie, Truth) mixed ANOVAs on the number of *omitted* details (see Figure 3, patterned bars) and the number of *new* details added in Phase II (see Figure 3, open bars). There was a main effect of Interview Technique on the number of details *omitted* from Phase II statements, *F*(1, 68) = 47.27, *p* < 0.001, *d* = 1.65 [1.10, 2.19]. Participants omitted more details from Phase II statements after being interviewed with a Structured Interview in Phase I (*M* = 20.16, *SE* = 2.00) relative to those interviewed with a Reverse Order instruction in Phase I (*M* = 4.84, *SE* = 0.51). However, there was no main effect of Veracity (*F*(1, 68) < 0.01, *p* = 0.93, η_p_^2^ < 0.01, nor an interaction between Veracity and Interview Technique, *F*(1, 68) = 0.10, *p* = 0.75, η_p_^2^ < 0.01. With respect to *new* details provided during Phase II, there was only a main effect of Interview Technique (*F*(1, 68) = 10.10, *p* < 0.01, *d* = 0.76 [0.27, 1.25]). Participants interviewed with a Structured Interview added more details in their Phase II statements (*M* = 5.74, *SE* = 0.62) than did participants interviewed with a Reverse Order instruction (*M* = 3.31, *SE* = 0.40). Neither the main effect of Veracity (*F*(1, 68) = 0.80, *p* = 0.37, *d* = 0.11 [−0.13, 0.34]), nor an interaction between the two (*F*(1, 67) = 1.74, *p* = 0.19, η_p_^2^ = 0.03.

### 3.2. Exploratory analysis

**Figure 4:**
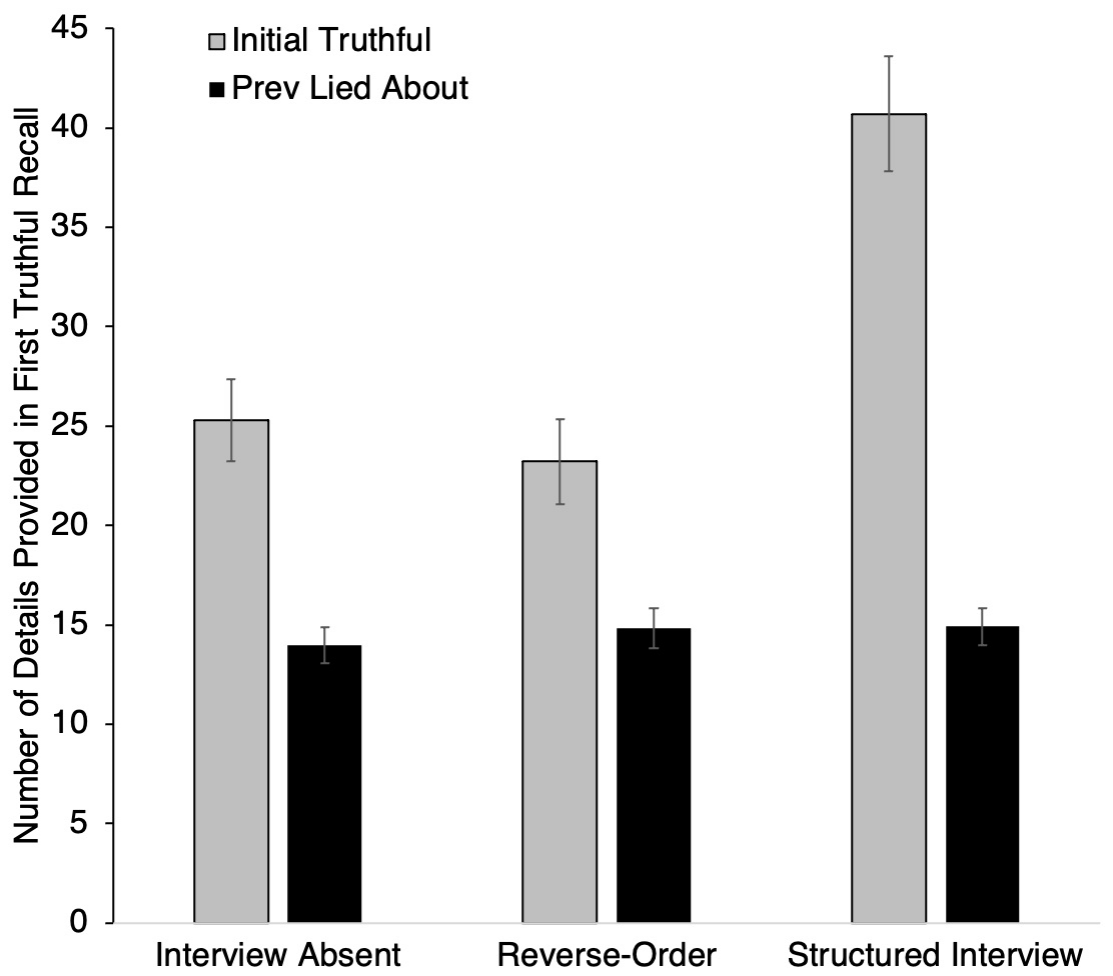
Number of details initially truthfully recalled per event area.

In addition to pre-registered analyses, we also explored how participants not interviewed at Phase I (Initial Interview-Absent) compared to participants who did receive an interview in Phase I with respect to differences in the amount of detail provided for the first time an area of campus was discussed (see Figure 4). For participants who received an initial interview, we examined whether the total amount of detail differed for their initial truthful statement (during the Phase I interview) relative to their truthful statement about the unrehearsed area of campus that they visited (during the Final All-Truth interview). For participants who were not interviewed during Phase I, we compared the amount of detail in their initial truthful statement (during the Phase II interview) to their truthful statement about the area of campus they visited that they did not rehearse previously (during the Final All-Truth interview).

Pairwise analyses were conducted to compare the amount of detail provided for the initial narrative about the previously lied-about event relative to the initial narrative about the previous truthfully rehearsed event for participants in each interview condition. Participants provided significantly more details when initially recalling their truthful event compared to when they truthfully recalled the event that they previously lied about in the Interview Absent condition (*t*(26) = 6.80, *p* < 0.001, *d* = 1.31 [0.78, 1.82]), in the Reverse Order condition (*t*(31) = 4.40, *p* < 0.001, *d* = 0.78 [0.38, 1.17]), and in the Structured Interview condition (*t*(36) = 9.55, *p* < 0.001, *d* = 1.57 [1.08, 2.05]).

## 4. Discussion

Given the frequency with which investigators repeatedly interview criminal suspects, the current research assessed whether a suspect’s choice to lie in an initial interview has consequences for memory accuracy and consistency on a subsequent interview. Here, we examined whether lying affects a suspect’s memory with respect to accuracy, as well as how credibility assessment interview techniques (such as the Reverse Order instruction) influence between-statement consistency. Our findings suggest that relative to lying, telling the truth led to better memory for encoded material and more consistent statements across interviews separated by a one-week delay. Further, those interviewed with a Structured Interview were more likely to omit details between two interview statements.

Experiences that participants had truthfully reported in an initial interview were associated with greater detail and were more accurately recalled when compared with those that were initially lied about. Regardless of whether or how they were interviewed during the initial session, participants provided more spontaneous accurate details in the final truthful interview when they had been previously truthfully interviewed compared to when they had previously lied about the experience. This aligns with previous findings (e.g., Battista et al., 2020; Dianiska and Meissner, 2022), and demonstrates that lying is detrimental to subsequent recall of the truth.

Here, the act of lying required participants to not only refrain from describing one area they visited, but also to create a false description of an area they had not visited. The mnemonic effect of lying seen here may thus be due to a relative lack of rehearsal, as suggested by the MAD framework (Otgaar and Baker, 2018). When participants provided false descriptions about an unvisited area of campus, they did so at the expense of not rehearsing an area of campus that was visited during the scavenger hunt. As a result, people provided less information about the unrehearsed (i.e., lied-about) area of campus when they were later asked to truthfully recall their experience (see Riesthuis et al., 2022b). The fact that the unrehearsed experience was associated with less detail could also be due to a spontaneous inhibition strategy that people may use to facilitate their lie-telling. That is, relative to areas that were truthfully rehearsed, in order to effectively produce a false description of an unexperienced event, people may have attempted to intentionally inhibit information about their activities in the unrehearsed area.

The content of people’s statements, both initially and in subsequent interviews, may serve to discriminate lies from truths. In the present experiment, truthfully provided statements about prior experiences were more detailed than experiences that were lied about. This was the case for statements obtained during both initial (Phase I) and delayed (Phase II) interviews. Consistent with prior research it is clear that the level of detail provided about an event can serve as an indicator of veracity (e.g., Evans et al., 2013). However, in the present study, people provided less detail on the Phase II interview for both truthful and deceptive statements after a one-week delay. That is, when providing their deceptive statements, people did not demonstrate the “stability bias” (i.e., similar detail across interviews for lied-about events) that has been observed by others with lengthier delays (i.e., three weeks; Harvey et al., 2017a,b). It may be the case that, at longer delays, the level of detail is a more effective indicator of whether a person is lying or telling the truth.

The nature of these details, such as whether they are consistent across time points, may also be important for the discrimination of lies and truths. Consistency and inconsistency across repeated interviews were considered with respect to four main types of information: repeated, omitted, reminiscent, and contradictory. Opportunities for repeated recall offer truth-tellers an occasion to appear inconsistent, should they provide new information in a subsequent statement. The addition of information that is reminiscent (and therefore inconsistent) may be more likely when people are cued to provide a second statement with a different cue than was used to elicit a prior statement (Gilbert and Fisher, 2006). Liars, on the other hand, may be perceived as suspicious should their statements be inconsistent across interviews and therefore may strategically attempt to maintain their narratives over time. Here, truthfully described activities were associated with a greater proportion of consistent details than were experiences that people lied about.

Asking participants to recall their activities in reverse chronological order actually *improved* between-statement consistency. Specifically, people in the Reverse Order condition omitted fewer details between Phase I and Phase II interviews, compared to people who were asked follow-up probing questions in the Structured Interview condition. Although we expected that participants interviewed with a Reverse Order technique would provide more detailed initial narratives, it was the “tell me more” probing questions in the Structured Interview that led to greater reported details–however, many of these details were subsequently omitted in the Phase II interview. Though accuracy for the details added following these probes could not be assessed for all statements (though other work suggests they may be less accurate than unprompted details; Kontogianni et al., 2020), it is likely that these additional details were peripheral to the primary tasks. For instance, some of these details reflected individuals that they saw (but presumably did not interact with; e.g., “there was a guy with big black glasses” and “I almost ran into a girl”), while others reflected their personal thought processes or observations during the task (e.g., “it was loud in there” and “I was too lazy to scan [a QR code on a flyer in Campus Area A] with my phone”). Therefore, one possibility is that the additional probes in the Structured Interview condition may have prompted less important or less memorable details in the initial interview, leading participants to fail to provide these details during a subsequent interview.

In contrast to expectations, participants were similarly detailed during their Phase II interviews regardless of whether they had been initially interviewed or not. This may be due to participants in the Interview-Absent conditions receiving their cover story information and preparation time immediately preceding their interviews at Phase II. However, this preparation time was needed to equate the instructions to those received by initially interviewed participants.

### 4.1. Limitations and applied implications

Though it may be possible for truth-tellers to be inconsistent in their repeated recall of an event, our findings suggest that the type of memory cuing afforded during the initial recall episode may be important. Contrary to our expectations, people did not provide more reminiscent details during the Phase II interview when truthfully describing their activities. However, this was likely due to the Phase II interview involving a simple free recall prompt rather than the use of memory-enhancing or varied retrieval approaches. As a result, any reminiscence would have been spontaneous (or self-cued). The use of a memory-enhancing technique, like the Cognitive Interview, has been shown to facilitate the reporting of new details in delayed recall (Odinot et al., 2013; Hope et al., 2014).

Both of the interviewing techniques used to elicit narratives in this study are considered “best practice.” The current research did not assess the effect of these best practice techniques in comparison to customary accusatorial tactics, such as those trained in the Reid technique (Inbau et al., 2011; see Meissner et al., 2015). Tactics that are characteristic of the Reid technique include shutting down denials, confronting the suspect with evidence of their guilt, and suggesting scenarios or theories of the crime. In future work, it may be useful to contrast the effects of lying on memory when best practice interview techniques are compared to such guilt-presumptive techniques.

Despite the benefit to some interview outcomes when “best practice” techniques are used (e.g., the diagnosticity of a confession; see Meissner et al., 2012, for a review), such techniques allow a subject to “tell their story” in a way that permits both denials as well as deceptive narratives. In a similar manner, approaches like the Cognitive Interview can lead to small increases in incorrect details being provided by the subject–though such interviews also lead to large increases in correct details, thereby mitigating the effect on a person’s overall accuracy (Memon et al., 2010). Could the provision of deceptive or incorrect information harm subsequent recall? The current data suggest that people who have previously lied are at a disadvantage should they decide at a later point to be truthful and forthcoming with an interviewer. We found that participants provided significantly less detail when they had previously lied about the event. What remains to be examined, however, is whether that harm might be partially or fully ameliorated if memory-enhancing techniques are used to elicit information in a later interview.

To motivate participants to lie convincingly during the experiment, we used a financial incentive based on others’ perceptions of their statement. Though participants rated their motivation to be perceived as truthful well above the midpoint of the scale (*M* = 5.43, *SD* = 0.99; on a scale from 1, not at all, to 7, completely), offering a monetary reward for believability might not have adequately motivated someone to lie as they might in an interview. As such, future work should investigate the effects of lying on memory when there is a stronger motivation to lie, such as to avoid punishment or embarrassment (e.g., Riesthuis et al., 2022a).

Finally, given the recent emphasis on increasing the ecological validity of deception experiments (Romeo et al., 2019; Dianiska and Meissner, 2022), participants in the present experiment were permitted to *choose* when to lie and when to tell the truth. Prior to being interviewed, all participants were tasked with choosing one area to tell the truth about and were then given an area of campus to create a lie about their activities. While the paradigm used in the present experiment offers more ecological validity than other lab-based paradigms, it does so at the expense of being able to assess participant’s statement accuracy. Given the variability in participants’ episodic experiences during the scavenger hunt (e.g., encountering different people and obstacles along the way), we could not assess accuracy. Future studies might involve the inclusion of a confederate or the use of a body camera in the experimental task that would allow for a more natural, yet verifiable, encoding task.

### 4.2. Conclusion

Taken together, the current findings add further evidence that the act of lying has downstream consequences for the accurate recall of truthfully experienced events. That is, lying about one’s experiences led to both less accurate memory for those experiences and less consistent statements. The current data suggest that the act of lying has a detrimental effect on memory for what truthfully occurred. Further, an interviewers’ choice of tactic can significantly influence the amount and quality of information provided. The use of a credibility assessment technique, such as the Reverse Order instruction, facilitated between-statement consistency by reducing omissions. In the absence of such a tactic, an interviewers’ selection of follow-up topics might, perhaps unintentionally, impede their ability to rely on consistency and the level of detail of a subject’s statement as cues to credibility.

## Data availability statement

The datasets presented in this study can be found in online repositories. The names of the repository/repositories and accession number(s) can be found at: https://osf.io/atz5h/.

## Ethics statement

The studies involving human participants were reviewed and approved by Iowa State University Institutional Review Board (IRB). The patients/participants provided their written informed consent to participate in this study.

## Author contributions

RD conceived the original idea and designed the study with contributions from CM, programmed, ran the experiment and analyzed the data, and wrote the primary drafts of the manuscript. CM assisted in analyses and interpretation of the data and provided feedback, and suggested revisions to the manuscript. All authors approved the submitted version of the manuscript.

## Conflict of interest

The authors declare that the research was conducted in the absence of any commercial or financial relationships that could be construed as a potential conflict of interest.

## Publisher’s note

All claims expressed in this article are solely those of the authors and do not necessarily represent those of their affiliated organizations, or those of the publisher, the editors and the reviewers. Any product that may be evaluated in this article, or claim that may be made by its manufacturer, is not guaranteed or endorsed by the publisher.

## Footnotes

^1^ https://osf.io/atz5h/
